# Nursing role central to successful implementation of enhanced recovery after surgery

**DOI:** 10.1016/j.apjon.2022.100112

**Published:** 2022-06-30

**Authors:** Gregg Nelson

**Affiliations:** Department of Obstetrics & Gynecology, University of Calgary, Calgary, Alberta, Canada

Enhanced recovery after surgery (ERAS) is a global surgical quality improvement initiative that is now firmly embedded as a standard of care in numerous surgical disciplines and is gaining traction in several others.^1^ A key component required for the successful implementation of ERAS is the development of a multidisciplinary team comprised at a minimum of a surgeon, an anesthesiologist, and a nurse.^2^ It is important to select these individuals carefully, as they will be the ones carrying out the work and promoting the uptake of ERAS at their local institution. The importance of nursing, not only the ERAS nurse in the core ERAS team but also the nurses in the clinic, operating room, recovery room and ward, cannot be overemphasized as they perform the bulk of the day-to-day tasks of the enhanced recovery protocol.^1^ Nurses also play a central role in getting teams to iterate toward increasing ERAS adherence which has been shown in several studies to be associated with improved clinical outcomes.^3^^,^^4^ Despite these efforts, many teams remain stalled at suboptimal ERAS adherence (< 70%) and lack of collaboration and communication between multidisciplinary team members has been identified as a major barrier.^5^^,^^6^ Are nurses the answer to improved outcomes in ERAS? Several articles in this issue of the *Journal* highlight the unique role that the nurse plays in ERAS implementation.

*Jensen et al*. performed a systematic review of efficacy of pre- and rehabilitation in radical cystectomy on health-related quality of life and physical function. While none of the included studies provided support that the interventions improved quality of life overall, the authors did find evidence that nursing led education in stoma care improved the self-efficacy significantly.^7^ Image-altering procedures such as stomas which are often employed in oncology surgeries (eg., urology, colorectal surgery, gynecologic oncology) require a thorough discussion with the patient, and this is often beyond the time allowable in clinic with the surgeon. This then falls to the experienced ERAS nurse who can explore the balance between therapeutic benefit and impact on quality of life for the patient. This is emphasized in *Jensen et al*'s ERAS nursing perspective in radical cystectomy, which describes the increasingly important role of shared decision making in oncology care, where ERAS nurses help patients faced with difficult treatment decisions that require them to weigh efficacy, safety, and quality of life.^8^

*Majumdar et al.* from Memorial Sloan Kettering Cancer Center implemented an updated ERAS protocol in total mastectomy patients which included a 35% increase in total intravenous anesthesia usage and near elimination of ketorolac. These changes resulted in a statistically significant reduction in reoperation due to hematoma formation.^9^ The authors attributed the protocol success to “use of effective feedback, communication, and teamwork translating to meaningful change in patient outcomes”.^9^ In another study, *Lu et al.* aimed to develop an ERAS protocol for lung cancer surgery tailored to the Chinese national context and extended their study search to Chinese databases including Chinese Biomedical Literature Database (Sinomed) and China Academic Journals (CNKI) among others.^10^ They are among the first to take this approach and contend that successful implementation of ERAS not only requires evidence-based practice but also must take into account the current status quo of the department and local patient conditions in order to develop a meaningful perioperative care plan. Undoubtedly, the successful outcomes seen in the ERAS mastectomy study^9^ and the roll-out of the Chinese-tailored ERAS lung protocol^10^ are reliant on the diligence of the ERAS nurse but also all nursing staff along the entire ERAS surgical care continuum as many of the ERAS recommendations relate to traditional nursing roles such as preoperative education, nutrition, mobilization, and analgesia.

Finally, it is now established that nursing workload actually decreases with the increasing ERAS compliance,^11^ mirroring the dose–response relationship seen with clinical outcomes (length of stay and complications) and ERAS compliance.^3^^,^^4^ Because of this, one might assume that the roles and responsibilities of the ERAS nurse should be ever increasing. This is not the case, however, according to *Balfour et al*. who discuss that twenty-five years on, we are still seeing barriers to expansion of the ERAS nurse role in today's hospitals.^12^ Barriers include poor nursing leadership, lack of engagement, poor communication, and lack of resources. Some nurses even fear reprisal for attempting to keep a patient on the ERAS pathway and question to what degree they will ever independently manage patients within the ERAS program. The answer will most likely vary from country to country but fortunately solutions to overcoming these barriers exist such as the inclusion of nursing in the ERAS Guidelines working groups and encouraging nurses to help generate the evidence base (research) that forms the backbone of the guidelines.

Nursing is central to the successful implementation of ERAS. The nursing role will continue to evolve within the ERAS program just as the ERAS guidelines themselves change and as ERAS teams innovate toward improvements in clinical outcomes for patients and healthcare systems globally.

## Declaration of competing interest

None declared.
